# Health-related quality of life in Iranian adolescents: a psychometric evaluation of the self-report form of the PedsQL 4.0 and an investigation of gender and age differences

**DOI:** 10.1186/s12955-021-01742-8

**Published:** 2021-03-26

**Authors:** Habib Hadianfard, Behnaz Kiani, Mahla Azizzadeh Herozi, Fatemeh Mohajelin, John T. Mitchell

**Affiliations:** 1 Department of Clinical Psychology, School of Education and Psychology, Shiraz University, Shiraz, Iran; 2 Department of Psychiatry and Behavioral Sciences, Duke University Medical Center, Durham, NC USA

**Keywords:** PedsQL 4.0, Quality of life, Internal consistency, Test–retest reliability, Construct validity, Item response theory (IRT), Convergent validity, Adolescents

## Abstract

**Background:**

Research on the psychometric properties of the Persian self-report form of the Pediatric Quality of Life Inventory Version 4.0 (PedsQL 4.0) in adolescents has several gaps (e.g., convergent validity) that limit its clinical application and therefore the cross-cultural impact of this measure. This study aimed at investigating the psychometric properties of the PedsQL 4.0 and the effects of gender and age on quality of life in Iranian adolescents.

**Method:**

The PedsQL 4.0 was administered to 326 adolescents (12–17 years). A subsample of 115 adolescents completed the scale two weeks after the first assessment. Confirmatory Factor Analysis (CFA), correlation of the PedsQL 4.0 with the Weiss Functional Impairment Rating Scale-Self-report (WFIRS-S), and Item Response Theory (IRT) analysis were conducted to examine validity. Cronbach’s alpha, McDonald’s Omega, and Intra class correlation (ICC) were calculated as well to examine reliability. Gender and age effects were also evaluated.

**Results:**

Internal consistency and test–retest reliability of the total PedsQL 4.0 scale was .92 and .87, respectively. The PedsQL 4.0 scores showed negative moderate to strong correlations with the WFIRS-S total scale. The four-factor model of the PedsQL 4.0 was not fully supported by the CFA—the root mean square error of approximation and the comparative fit index showed a mediocre and poor fit, respectively. IRT analysis indicated that all items of the PedsQL 4.0 fit with the scale and most of them showed good discrimination. The items and total scale provided more information in the lower levels of the latent trait. Males showed significantly higher scores than females in physical and emotional functioning, psychosocial health, and total scale. Adolescents with lower ages showed better quality of life than those with higher ages in all scores of the PedsQL 4.0.

**Conclusion:**

The PedsQL 4.0 showed good psychometric properties with regard to internal consistency, test–retest reliability, and convergent validity in Iranian adolescents, which supports its use in clinical settings among Persian-speaking adolescents. However, factor structure according to our CFA indicates that future work should address how to improve fit. In addition, studies that include PedsQL 4.0 should consider gender and age effects were reported.

## Background

Health-related quality of life refers to one's perception and subjective appraisal of his/her health and well-being within a cultural context [1]. According to the World Health Organization [2], health is not just the absence of disorder and weakness, but the presence of physical, mental, and social well-being. Given these definitions, health encompasses well-being in different domains of functioning and quality of life includes well-being in those domains.

Health-related quality of life has been recognized as an important outcome measure in health care services and clinical decisions. There are various measures for assessing health-related quality of life and one of these measures is the Pediatric Quality of Life Inventory, Version 4.0 (PedsQL 4.0) [3]. The PedsQL 4.0 is a multidimensional instrument measuring physical, emotional, social, and school functioning that has been translated into numerous languages. This scale consists of child self-report forms for ages 5–7, 8–12, and 13–18 years and parent proxy-report forms for ages 2–4 (toddler), 5–7 (young child), 8–12 (child), and 13–18 (adolescent) [4]. The Persian version of the PedsQL 4.0 has been psychometrically evaluated in several studies on healthy and patient samples of Iranian children and adolescents [5–9]. Among these studies, one study [5] used a sample of children and adolescents diagnosed with Type 1 Diabetes and their parents. The sample in two studies [6, 7] included healthy and chronically ill children and their parents. Participants in one study [8] consisted of a group of children with attention-deficit/hyperactivity disorder (ADHD) and their parents and a school-based control group with their parents. The sample of one study [9] consisted of school children and their parents. While these studies make important contributions, to our knowledge, only one study [7] has assessed the psychometric properties of the Persian self-report form of the PedsQL 4.0 in adolescents. Moreover, no previous study has examined the convergent validity of the PedsQL 4.0 in Iranian adolescents. Additionally, only one study [7] has assessed and compared the PedsQL 4.0 scores between male and female adolescents ages 13–18 and no study has addressed the age differences in PedsQL 4.0 scores in Iranian adolescents. In Amiri et al. [7], the PedsQL 4.0 self-report form showed construct validity and good internal consistency for the subscales (r = 0.68 to 0.78) and for the total scale (r = 0.88). However, convergent validity, test–retest-reliability, and age differences in the PedsQL 4.0 were not assessed. There is a need for studies to more fully examine the psychometric properties of the Persian self-report form of the PedsQL 4.0 in adolescents to improve real-world assessment in clinical and school settings.

The first aim of this study is to assess the validity and reliability of the Persian self-report form of the PedsQL 4.0 in a healthy sample of Iranian adolescents. The second aim of this study is to investigate the effects of gender and age on the PedsQL 4.0 scores. Given the finding that among Iranian adolescents, males have better quality of life than females in the physical and emotional subscales, and total scale of the PedsQL 4.0 [7], we hypothesized that males will show better quality of life in physical functioning, emotional functioning, and total scale of the PedsQL 4.0.

## Method

### Procedure

Participants were taken from four public secondary schools through multistage sampling. All participants provided assent and their parents provided written consent. Participants received information about the aim of the study and necessary instructions for answering the questionnaires. In order to assess the test–retest reliability, a subsample of 115 participants completed the PedsQL 4.0 two weeks after the first administration. These participants were selected from classes that had enough time to allow students participate in retest evaluation. Students completed the questionnaires in a classroom setting. Inclusion criteria were ages ≥ 12 to ≤ 17 years old and enrolled in grades 7 to 12. All students with the mentioned criteria were eligible to be included in this study. Psychopathology was not assessed and was not part of the study inclusion criteria. This study was approved by the Research Ethics Committee of School of Psychology of Shiraz University and also by the Research Ethics Committee of the Shiraz School Board. This study was done in accordance with the ethical codes of the Psychology and Counseling Organization of Iran.

### Measures

#### Pediatric quality of life inventory version 4.0-self-report (PedsQL 4.0)

In this study, the self-report form of the PedsQL 4.0 was used for all age groups. The PedsQL 4.0 includes 23 items covering four subscales: physical functioning (8 items, e.g., "It is hard for me to do sports activity or exercise"), emotional functioning (5 items, e.g., "I feel sad or blue"), social functioning (5 items, e.g., "Other kids don't want to be my friend"), and school functioning (5 items, e.g., "I have trouble keeping up with my schoolwork"). In addition to computing the score of each of the mentioned subscales, psychosocial health summary score was obtained by calculating the sum of item scores in the emotional, social, and school functioning subscales divided by the number of responded items [10]. The respondent is instructed to rate the severity of his/her problem in each item based on a 5-point Likert response scale (0 = never a problem; 1 = almost never a problem; 2 = sometimes a problem; 3 = often a problem; 4 = almost always a problem) during the last month. The scores are reversed based on a 0 to 100 scale (0 = 100, 1 = 75, 2 = 50, 3 = 25, and 4 = 0) and higher scores indicate better quality of life.

#### Weiss functional impairment rating scale-self-report (WFIRS-S)

The WFIRS-S [11] was developed to measure the impact of emotional and behavioral problems on functional impairment. The WFIRS-S includes 69 items assessing an adolescent’s or an adult’s functioning across seven domains: family (8 items, e.g., "Problems taking care of your family"), work (11 items, e.g., "Problems working in a team"), school (10 items, e.g., "Problems meeting minimum requirements to stay in school" life skills (12 items, e.g., "Problems with sleeping"), self-concept (5 items, e.g., "Feeling incompetent"), social (9 items, e.g., "Problems making friends") and risk (14 items, e.g., "Being involved with the police"). The items can be rated on a 0 (never or not at all) to 3 (very often or very much) Likert scale or can be rated as “not applicable”. The WFIRS-S has been shown to be a valid and reliable rating scale in measuring functional impairment [12]. The Persian version of the WFIRS-S has been psychometrically assessed in Iranian adolescents and the internal consistency and test–retest reliability were respectively 0.94 and 0.80 for the total scale [13].

### Data analyses

Statistical analyses were performed using IBM SPSS Statistics 22, IBM SPSS Amos 24, Stata 14, and IRTPRO 5.0.

#### Descriptive statistics

Mean, standard deviation, median, skewness, and kurtosis were determined for the PedsQL 4.0 items.

#### Reliability

Internal consistency of each subscale and total scale of the PedsQL 4.0 was assessed by Cronbach’s alpha and McDonald’s Omega [11]. Internal consistency is considered acceptable, good, and excellent for Cronbach’s alpha coefficients greater than 0.7, 0.8, and 0.9, respectively [14]. The Cronbach’s alpha requires the assumption of tau-equivalence [15] and shows bias in estimating the internal consistency for Likert type rating response scales [16], therefore, McDonald’s Omega was also estimated to evaluate the internal consistency. McDonald’s Omega values above 0.7 and 0.8 can be interpreted as acceptable and good estimates of internal consistency, respectively [17, 18]. Test–retest reliability was measured by two-way random effects model, absolute agreement intraclass correlation coefficient (ICC) [19]. ICC between 0.61–0.80 is interpreted as a good reliability and between 0.81–1.00 is considered excellent [20].

#### Validity

Construct validity of the PedsQL 4.0 was assessed in this study. Construct validity is defined as the extent to which an instrument assesses the hypothesis or theory it aimed to measure [21]. Different components of the construct validity including convergent validity and factorial validity were examined. Moreover, Item Response Theory (IRT) as a modern psychometric method was performed for assessing the construct validity. Convergent validity of the PedsQL 4.0 with the WFIRS-S was measured by computing Spearman’s rank correlation coefficient. We used WFIRS-S to measure the convergent validity of the PedsQL 4.0 for two reasons. First, the WFIRS-S measures functional impairment which has been considered in conceptualization of the quality of life [12]. Second, the previous studies [13, 22] showed moderate to strong correlations between the PedsQL 4.0 and WFIRS that we expected this correlation size in our study. Because higher scores in the PedsQL 4.0 indicate better quality of life and lower scores in the WFIRS-S show better functioning in life domains, negative correlations between the scores of two scales was expected. Correlation coefficients below 0.29 is considered small, between 0.30 and 0.49 is moderate, and greater than 0.50 is high [23]. In order to assess the factorial validity of the PedsQL 4.0, Confirmatory Factor Analysis (CFA) was conducted. For the CFA, the four-factor model of the scale confirmed in other studies [24, 25] was assessed. The goodness of fit of the CFA model was examined by using the comparative fit index (CFI) and the root mean square error of approximation (RMSEA). Rigdon [26] argues that the RMSEA appears to be a better index in confirmatory contexts, while CFI is an appropriate index in exploratory contexts. CFI [27] is an incremental fit index which is based on the comparison of a hypothesized model with a null model and ranges between 0 (poor fit) and 1.00 (perfect fit). A CFI ≥ 0.95 shows a good fit [28]. RMSEA [29] is one of the absolute measures of fit which determines how well a hypothesized model fits a perfect model. A value of RMSEA < 0.08 indicates an appropriate fit and a value < 0.1 indicates a mediocre fit [30]. For the IRT evaluation, the graded response model (GRM) was applied [31]. GRM is appropriate when dealing with ordered polytomous items such as Likert response items. For each item, the goodness-of-fit index (the *p*-value of S-χ2 < 0.001) [32, 33], discrimination parameter, and threshold or difficulty parameter were calculated. Discrimination parameter refers to the ability of an item in distinguishing between different levels of the latent trait. Threshold or difficulty parameter demonstrates the level of the latent trait in which the probability of response to or above a given category equals 0.5. Category characteristic curve and item information curve for each item and test information curve for the total scale of the PedsQL 4.0 were analyzed. Category characteristic curve represents the probability of responding to a given category of an item as a function of the latent trait. Information curve indicates the amount of information provided by an item or a scale at various levels of the latent trait. The discrimination parameter of an item determines the height of the information curve and the threshold parameter(s) determines the location of the information curve. Higher information curve shows more precision for an item in measuring the latent trait.

#### Age and gender differences

Kruskal–Wallis test was used to assess the effects of gender and age on the PedsQL 4.0 subscales, psychosocial health scale, and total scale. Dunn’s test was performed for pairwise comparisons. A *p*-value less than 0.05 was regarded as statistically significant.

## Results

### Participants

The sample of the current study were 326 male and female adolescents (mean age = 14.84, SD = 1.78) who were studied in grades 7 to 12 of public secondary schools of Shiraz, Iran (see Table 1 for more details).

**Table 1:** participants characteristics (n = 326)

Characteristic	
Age (M (SD))	14.84 (1.78)
Gender (n (%))	
Male	159 (48.8%)
Female	167 (51.2%)
Grade (n (%))	
Seventh	63 (19.3%)
Eighth	51 (15.6%)
Ninth	48 (14.7%)
Tenth	53 (16.3%)
Eleventh	62 (19.0%)
Twelfth	49 (15.1%)
Grade by age group (years) equivalent	
Seventh	12
Eighth	13
Ninth	14
Tenth	15
Eleventh	16
Twelfth	17

*M* mean, *SD* standard deviation

### Descriptive statistics

Table 2 shows the mean, standard deviation, median, skewness, and kurtosis for the items of the PedsQL 4.0. For the physical functioning items, the mean scores ranged from 79.31 (SD = 27.37) to 96.70 (SD = 12.96). The mean scores of the emotional functioning items were between 75.40 (SD = 29.31) and 85.63 (SD = 25.97). For the social functioning items, the mean scores varied in the range of 89.23 (SD = 20.68) and 93.71 (SD = 16.25). The mean scores of the school functioning items were between 79.09 (SD = 25.59) and 86.81 (SD = 22.38). For all items of the PedsQL 4.0, the skewness was lower than 1 except for item 4 of the emotional functioning with a skewness of − 0.84. All items of the physical functioning except for item 4, item 4 of the emotional functioning, all items of the social functioning, and all items of the school functioning except for item 2 showed a kurtosis higher than 1.

**Table 2:** Descriptive statistics of the PedsQL 4.0 items (n = 326)

	Mean	SD	Median	Skewness	Kurtosis
Physical functioning					
Item 1	87.08	23.04	100	− 1.72	2.18
Item 2	83.05	25.33	100	− 1.40	1.11
Item 3	87.35	23.15	100	− 1.70	1.79
Item 4	79.31	27.37	100	− 1.06	0.05
Item 5	96.70	12.96	100	− 4.71	24.93
Item 6	88.20	21.63	100	− 1.80	2.44
Item 7	88.81	21.55	100	− 2.09	4.11
Item 8	86.92	22.70	100	− 1.76	2.59
Emotional functioning					
Item 1	84.60	23.25	100	− 1.35	0.96
Item 2	79.38	26.90	100	− 1.01	− 0.10
Item 3	79.49	27.29	100	− 1.11	0.24
Item 4	85.63	25.97	100	− 1.85	2.56
Item 5	75.40	29.31	100	− .84	− 0.43
Social functioning					
Item 1	90.95	18.04	100	− 2.10	3.82
Item 2	93.71	16.25	100	− 3.35	13.35
Item 3	89.23	20.68	100	− 2.02	3.68
Item 4	93.19	18.06	100	− 3.22	10.97
Item 5	91.13	19.88	100	− 2.59	6.90
School functioning					
Item 1	83.93	23.09	100	− 1.44	1.75
Item 2	79.09	25.59	75	− 1.17	0.85
Item 3	82.38	25.77	100	− 1.42	1.28
Item 4	86.81	22.38	100	− 1.81	2.92
Item 5	82.04	25.36	100	− 1.38	1.33

PedsQL 4.0 = Pediatric quality of life inventory version 4.0

*SD* standard deviation

### Internal consistency

The Cronbach’s alpha was good (α = 0.80–0.83) for the subscales of the PedsQL 4.0 and was excellent for the psychosocial health (α = 0.90) and total scale (α = 0.92) of the PedsQL 4.0. The McDonald's Omega was good for the subscales (ω_t_ = 0.81 to 0.84), psychosocial health (ω_t_ = 0.90), and total scale (ω_t_ = 0.92) of the PedsQL 4.0 (Table 3).

**Table 3:** Internal consistency (n = 326) and test–retest reliability (n = 115) for the PedsQL 4.0

PedsQL 4.0	Number of Items	Internal consistency		Test–retest reliability
		α	ω_t_	ICC
Physical functioning	8	0.82	0.83	0.80
Emotional functioning	5	0.83	0.84	0.88
Social functioning	5	0.80	0.81	0.86
School functioning	5	0.81	0.81	0.74
Psychosocial health	15	0.90	0.90	0.87
Total score	23	0.92	0.92	0.87

PedsQL 4.0 = Pediatric quality of life inventory version 4.0

α = Cronbach’s alpha

ω_t_ = McDonald’s Omega

*ICC* intraclass correlation coefficient

### Test–retest reliability

As shown in Table 3, the test–retest reliability was good for the school functioning (ICC = 0.74) and was excellent for the physical functioning, emotional functioning, social functioning, psychosocial health, and total scale of the PedsQL 4.0 (ICCs = 0.80–0.88).

### Convergent validity

Correlations between the PedsQL 4.0 and the total scale of the WFIRS-S indicated that physical and social functioning subscales had moderate and significant correlations with the WFIRS-S total scale (*r* = − 0.44 and *r* = − 0.48, respectively; *p* = 0.001). Emotional and school functioning subscales, psychosocial health summary score, and total scale of the PedsQL 4.0 showed high and significant correlations with the WFIRS-S total scale (*r* = − 0.54 to − 0.62; *p* = 0.001).

### CFA

The results of CFA for the four-factor model of the PedsQL 4.0 showed that the RMSEA was 0.08 and the CFI was 0.84.

### IRT evaluation

Table 4 demonstrates the goodness-of-fit index, the discrimination parameter, and the threshold parameter for 23 items of the PedsQL 4.0. The *p*-value of S-χ2 showed that all items fit the scale.

**Table 4:** Discrimination parameter, threshold indices, and goodness-of-fit index for the items of the self-report form of the PedsQL 4.0

	Discrimination	Threshold indices				S-χ^2^	*p*
	a	b1	b2	b3	*b*4		
Physical functioning							
Item 1	1.19	− 4.18	− 3.08	− 1.65	− 1.01	55.63	0.16
Item 2	1.83	− 2.79	− 1.98	− 1.12	− 0.46	68.09	0.15
Item 3	2.03	− 3.20	− 2.01	− 1.23	− 0.83	44.78	0.48
Item 4	1.45	− 3.15	− 2.08	− 0.95	− 0.32	79.84	0.09
Item 5	2.01	− 3.25	− 3.04	− 2.22	− 1.83	23.28	0.03
Item 6	2.10	− 3.21	− 2.19	− 1.32	− 0.78	43.42	0.37
Item 7	2.08	− 2.74	− 2.29	− 1.41	− 0.83	45.48	0.19
Item 8	3.02	− 2.37	− 1.95	− 1.10	− 0.63	42.50	0.41
Emotional functioning							
Item 1	2.07	− 3.02	− 2.17	− 1.06	− 0.49	63.10	0.08
Item 2	1.99	− 2.81	− 1.73	− 0.82	− 0.28	54.99	0.48
Item 3	2.46	− 2.29	− 1.62	− 0.81	− 0.28	56.00	0.26
Item 4	1.90	− 2.50	− 1.90	− 1.33	− 0.76	66.37	0.04
Item 5	2.29	− 2.21	− 1.41	− 0.56	− 0.08	69.91	0.16
Social functioning							
Item 1	1.64	− 2.79	− 1.96	− 1.03		22.00	0.97
Item 2	1.82	− 3.14	− 2.19	− 1.32		36.57	0.05
Item 3	2.28	− 2.86	− 2.27	− 1.40	− 0.83	38.47	0.45
Item 4	2.25	− 2.72	− 2.25	− 1.85	− 1.18	36.67	0.13
Item 5	2.59	− 2.49	− 2.19	− 1.49	− 0.96	39.00	0.18
School functioning							
Item 1	2.72	− 2.33	− 2.08	− 1.03	− 0.36	46.38	0.50
Item 2	2.28	− 2.32	− 1.85	− 0.94	− 0.06	60.29	0.17
Item 3	2.56	− 2.28	− 1.71	− 1.00	− 0.36	52.22	0.18
Item 4	1.08	− 4.27	− 3.32	− 1.90	− 0.83	56.98	0.33
Item 5	1.09	− 3.67	− 3.04	− 1.39	− 0.40	95.26	0.009

PedsQL 4.0 = Pediatric quality of life inventory version 4.0

In the physical functioning subscale, the discrimination parameter of the items ranged from 1.19 to 3.2 (Table 4). Item 8 was the highest discriminating item (*a* = 3.02) and item 1 was the lowest discriminating item (*a* = 1.19). In terms of the severity (Table 4), item 1 (*b*_*1*_ = − 4.18, *b*_*2*_ = − 3.08, *b*_*3*_ = − 1.65, *b*_*4*_ = − 1.01) and item 5 (*b*_*1*_ = − 3.25, *b*_*2*_ = − 3.04, *b*_*3*_ = − 2.22, *b*_*4*_ = − 1.83) emerged at lower levels of the latent trait (physical functioning) and item 2 (*b*_*1*_ = − 2.79, *b*_*2*_ = − 1.98, *b*_*3*_ = − 1.12, *b*_*4*_ = − 0.46) and item 8 (*b*_*1*_ = − 2.37, *b*_*2*_ = − 1.95, *b*_*3*_ = − 1.10, *b*_*4*_ = − 0.63) were endorsed at higher levels of the latent trait (physical functioning).

In the emotional functioning subscale, the discrimination parameter of the items ranged from 1.90 to 2.46 (Table 4). Item 3 was the highest discriminating item (a = 2.46) and item 4 was the lowest discriminating item (a = 1.90). In terms of the severity (Table 4), item 1 (*b*_*1*_ = − 3.02, *b*_*2*_ = − 2.17, *b*_*3*_ = − 1.06, *b*_*4*_ = − 0.49) emerged at lower levels of the latent trait (emotional functioning) and item 5 (*b*_*1*_ = − 2.21, *b*_*2*_ = − 1.41, *b*_*3*_ = − 1.56, *b*_*4*_ = − 0.08) was endorsed at higher levels of the latent trait (emotional functioning).

In the social functioning subscale, the discrimination parameter of the items ranged from 1.64 to 2.59 (Table 4). Item 5 was the highest discriminating item (a = 2.59) and item 1 was the lowest discriminating item (a = 1.64). For items 1 and 2, only three threshold indices were obtained because the frequency of response to the first category for item 1 and second category for item 2 was zero. In terms of the severity (Table 4), items emerged at similar levels of the latent trait (social functioning).

In the school functioning subscale, the discrimination parameter of the items ranged from 1.08 to 2.72 (Table 4). Item 1 was the highest discriminating item (a = 2.72) and item 4 was the lowest discriminating item (a = 1.08). In terms of the severity (Table 4), item 4 (*b*_*1*_ = − 4.27, *b*_*2*_ = − 3.32, *b*_*3*_ = − 1.90, *b*_*4*_ = − 0.83) emerged at lower levels of the latent trait (school functioning) and item 2 (*b*_*1*_ = − 2.32, *b*_*2*_ = − 1.85, *b*_*3*_ = − 0.94, *b*_*4*_ = − 0.06) was endorsed at higher levels of the latent trait (school functioning).

**Fig. 1:**
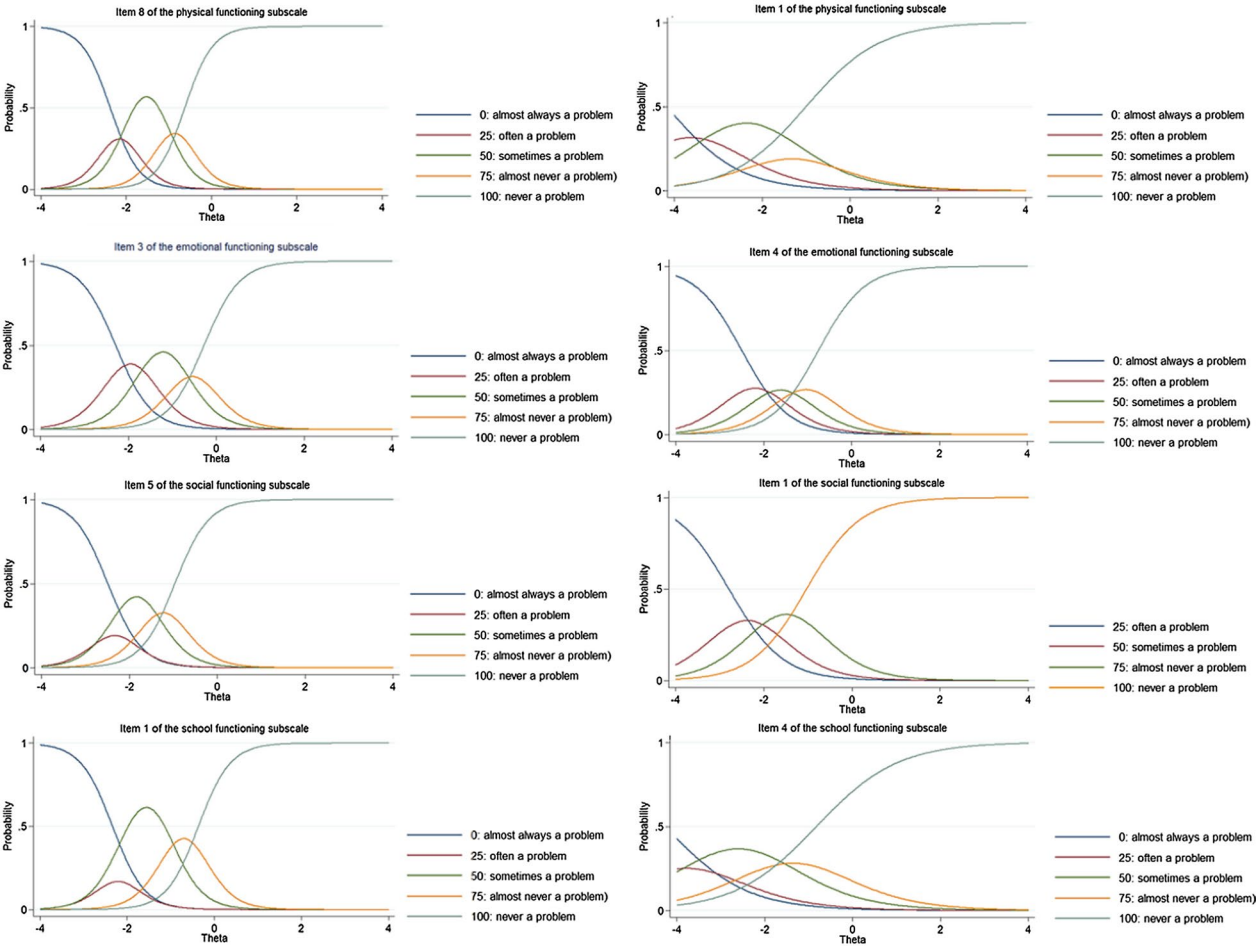
Category Characteristic Curves for the highest discriminating item (item 8 of the physical functioning, item 3 of the emotional functioning, item 5 of the social functioning, and item 1 of the school functioning) and the lowest discriminating item (item 1 of the physical functioning, item 4 of the emotional functioning, item 1 of the social functioning, and item 4 of the school functioning) in each subscale of the PedsQL 4.0

Figure 1 shows the category characteristic curves for the highest discriminating item and the lowest discriminating item of each subscale of the PedsQL 4.0. As can be observed in the category characteristic curves, the items generally were endorsed in below average levels of the latent trait which shows lower levels of quality of life.

**Fig. 2:**
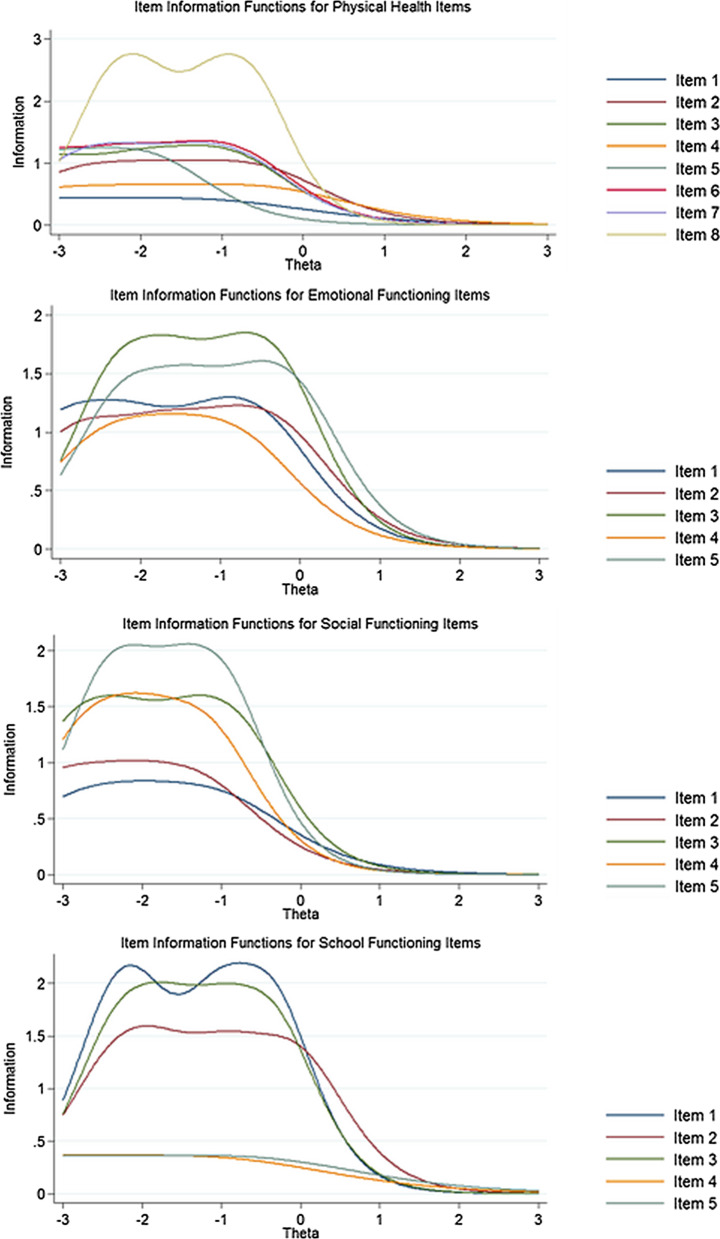
Item Information Functions for the physical, emotional, social, and school functioning subscales and Test Information Function for the total scale of the PedsQL 4.0

**Fig. 3:**
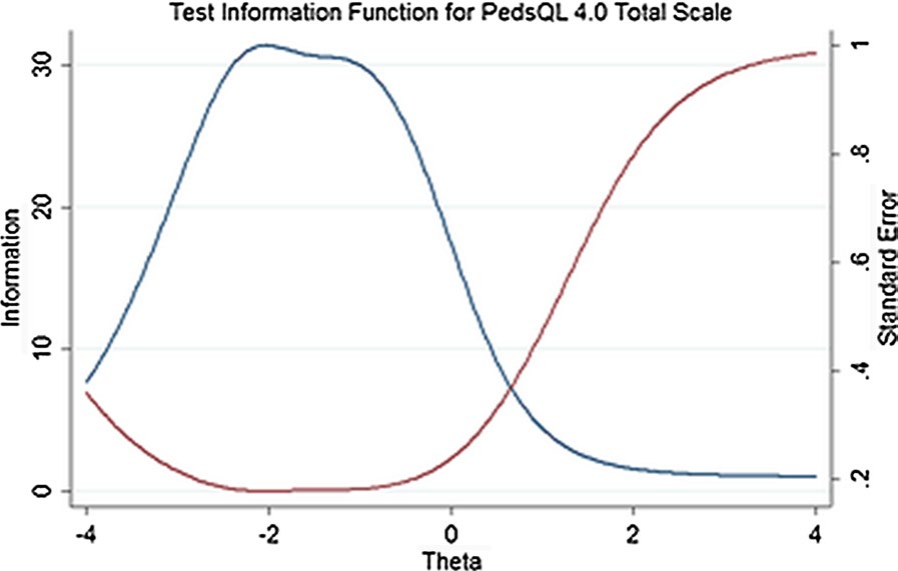
Test Information Function for the total scale of the PedsQL 4.0

Figure 2 shows the item information functions for the items of each subscale of the PedsQL 4.0. Item 8 in the physical functioning subscale, items 3 and 5 in the emotional functioning subscale, items 5 and 3 in the social functioning subscale, and items 1 and 3 in the school functioning subscale provided more information than other items. Figure 3 shows the test information function for the PedsQL 4.0 total scale. As can be seen in Fig. 3, the items of the PedsQL 4.0 provided more information for the lower levels of latent trait with the range of − 2.4 to − 0.8.

### Gender and age effects on PedsQL 4.0 scores

Table 5 shows the results of the Kruskal–Wallis test to compare males and females in the PedsQL 4.0 scores. There were significant differences between males and females in Physical Functioning (*χ*^*2*^(1) = 25.76,* p* = 0.001) and Emotional Functioning (*χ*^*2*^(1) = 22.81,* p* = 0.001). Male adolescents showed higher mean rank scores (better quality of life) than females in the physical functioning and emotional functioning subscales. Significant effects were found between males and females in the psychosocial health scale (*χ*^*2*^(1) = 9.97,* p* = 0.002) the total scale (*χ*^*2*^(1) = 13.89,* p* = 0.001). Male adolescents showed higher mean rank scores than females on the total scale (Table 5).

**Table 5:** Gender differences in the PedsQL 4.0 domains and total scale in adolescents (n = 326)

	Male (n = 159)	Female (n = 167)	χ^2^(1)	*p*
	Mean rank	Mean rank		
Physical functioning	189.99	138.28	25.76	0.001
Emotional functioning	188.52	139.68	22.81	0.001
Social functioning	168.76	158.49	1.17	0.28
School functioning	171.38	156.00	2.25	0.13
Psychosocial health	180.30	147.50	9.97	0.002
Total score	183.39	144.56	13.89	0.001

PedsQL 4.0 = Pediatric quality of life inventory version 4.0

Table 6 shows the results of the Kruskal–Wallis test to compare age groups in the PedsQL 4.0 scores. There were significant differences in physical functioning (*χ*^*2*^(5) = 18.53,* p* = 0.002), emotional functioning (*χ*^*2*^(5) = 28.83,* p* = 0.001), social functioning (*χ*^*2*^(5) = 16.54,* p* = 0.005), school functioning (*χ*^*2*^(5) = 29.57,* p* = 0.001) subscales, psychosocial health scale (*χ*^*2*^(5) = 34.86,* p* = 0.001), and total scale (*χ*^*2*^(5) = 34.71,* p* = 0.001) of the PedsQL 4.0 among ages (Table 6). Pairwise comparisons indicated that adolescents in lower ages have significantly higher mean scores in all subscales, psychosocial health, and total scale of the PedsQL 4.0 than adolescents in higher ages (Table 6).

**Table 6:** Age differences in the PedsQL 4.0 domains and total scale in adolescents (n = 326)

	Age						χ^2^(5)	Pairwise comparisons
	12 (n = 63)	13 (n = 51)	14 (n = 48)	15 (n = 53)	16 (n = 62)	17 (n = 49)		
	Mean rank	Mean rank	Mean rank	Mean rank	Mean rank	Mean rank		
Physical functioning	195.24	178.47	177.42	145.58	141.37	140.87	18.53**	12 > 16, 17**
Emotional functioning	203.06	179.83	179.40	148.42	143.02	122.29	28.83**	13, 14 > 17*; 12 > 16, 17**; 12 > 15*
Social functioning	191.87	163.44	181.16	159.70	144.77	137.60	16.45**	12 > 16, 17*
School functioning	208.86	172.41	174.26	143.02	151.73	122.41	29.57**	12 > 15, 16, 17**
Psychosocial health	209.59	178.20	180.43	148.30	140.86	117.45	34.86**	13, 14 > 17*; 12 > 16, 17**
Total score	208.98	180.94	180.75	147.11	137.78	120.24	34.71**	13, 14 > 17*; 12 > 15, 16, 17**

PedsQL 4.0 = Pediatric quality of life inventory version 4.0

*p < 0.05, **p < 0.01

## Discussion

This study assessed the psychometric properties of the Persian version of the PedsQL 4.0 self-report form in Iranian adolescents ages 12–17. The gender and age effects on the PedsQL 4.0 scores were examined. Internal consistency was good to excellent—across both methods of measurement (i.e., Cronbach’s alpha coefficient and McDonald’s omega)—for the subscales and total scale of the PedsQL 4.0. Internal consistencies in this study are similar to the results in Varni, Burwinkle, Seid, and Skarr’s study [34] and higher than in another study [7] in Iranian adolescents with a similar age (13–18 years) to our sample. In this study, we also assessed test–retest reliability, convergent validity of the PedsQL 4.0 with a measure of performance in functional domains of life (i.e., WFIRS-S), and the age differences in the PedsQL 4.0 scores—these outcomes have not been examined in previous studies of a Persian-speaking sample and are discussed in greater detail below.

Descriptive investigation of the PedsQL 4.0 items showed that the mean score for most items was close to 80 or above this score. All items showed a negative skewness. These findings show that the participants normally report no major problem in most items of the PedsQL 4.0. These results could be expected in healthy school-based samples and are consistent with the results of previous studies with similar samples [7, 24].

This is the first study reporting the test–retest reliability of the PedsQL 4.0 specifically in an Iranian sample of adolescents ages 12–17 years. Test–retest reliability was 0.87 for the total scale and psychosocial health and between 0.74 and 0.88 for the subscales. In a study of Iranian children and adolescents ages 8–18 years, test–retest reliability of the self-report form of the PedsQL 4.0 was 0.87 for the total scale and 0.71 to 0.80 for the subscales [9]. The difference between the results of test–retest reliability for the subscales of the PedsQL 4.0 in the current study and the results of Pakpour et al.’s study [9] might be explained by the age difference between the sample of two studies.

The current study is the first to assess the convergent validity of the PedsQL 4.0 with another measure in Iranian adolescents. Correlation between the psychosocial health score of the PedsQL 4.0 and functional impairment on the WFIRS-S was negative and high. The correlation between physical functioning and functional impairment was negative and moderate. The stronger correlation of functional impairment with psychosocial health score than with physical functioning reflects more overlap between the PedsQL 4.0 emotional, social, and school functioning subscales with the WFIRS-S content.

The findings of CFA showed that the fit of the four-factor structure of the PedsQL 4.0 was mediocre according to the RMSEA and poor based on CFI. This finding shows that the four-factor model of the PedsQL 4.0 was not fully supported by the CFA indices. The four-factor structure of the PedsQL 4.0 was confirmed in Kook and Varni’s [24] study in which a larger sample of children with a broader age range (8–18 years) was used in comparison to our study. The results of Varni et al.’s study [35] showed that the four-factor model of the PedsQL 4.0 was acceptable but the five-factor model including physical, emotional, social, school, and missed school factors indicated a marginally superior fit relative to the four-factor model.

The results of the IRT analysis showed that all items of the PedsQL 4.0 had good fitness with the scale. The discrimination parameter indicated that most items of the PedsQL 4.0 were highly discriminating (a = 1.45–3.02) [36]. Among the PedsQL 4.0 items, item 8 of the physical functioning, item 3 of the emotional functioning, item 5 of the social functioning, and item 1 of the school functioning showed the highest discrimination and items 1 and 4 of the physical functioning and items 4 and 5 of the school functioning showed the lowest discrimination. Lower discriminating power for items 4 and 5 of the school functioning was also found in another study by Hill et al. [37]. Items 4 and 5 assessed the degree to which a respondent misses school because of not feeling well or to go to the doctor/hospital. The events proposed in these items for missing the school are more frequent in populations with chronic disorders [37] not in healthy individuals that consists our sample. Therefore, these items provided low information and were less precise for measuring quality of life in our sample. The evaluation of the threshold parameter demonstrated that all items of the PedsQL 4.0 were located at the lower half of the latent trait. The evaluation of the information provided by each item of the PedsQL 4.0 and by the total scale indicated that this measure would be more precise and useful for measuring the lower levels of quality of life.

The investigation of gender and age effects on the PedsQL 4.0 scores showed that males perceived their function as better in physical and emotional domains relative to female adolescents. This finding is consistent with studies conducted in other countries [38–40] and with a study on Iranian adolescents [7]. Higher rated physical functioning in males than females may reflect a gender-based disparity in physical strength. Moreover, lower physical functioning and emotional functioning in females than males may have been caused by gender differences in mood problems. Females are not only more vulnerable to have depression, but also, they are more seriously affected by chronic depression, for example they show lower levels of quality of life when they have chronic depression [41]. Epidemiological studies have indicated that females report more internalizing problems than males [42, 43]. Our findings related to gender differences in quality of life can also demonstrate the sensitivity of the PedsQL 4.0 to identify these differences. Our cross-sectional study of age on quality of life showed that older adolescents reported more difficulties in all domains of quality of life relative to adolescents with the lower ages. Adolescence is an intermediate developmental stage between childhood and adulthood, and represents a phase of life involving diverse physical, psychological, and social changes and experiences that could influence health-related quality of life [44].

### Limitations

In this study, health-related quality of life was measured by adolescents’ self-report. Future studies should assess the test–retest reliability of the PedsQL 4.0 in adolescents by using parent proxy-report. In this study, only one measure was used to examine the convergent validity of the PedsQL 4.0. Future studies should consider additional measures to assess the convergent validity of the PedsQL 4.0. Another limitation of our study was that the same reporting source (i.e., self-report) was used for our outcome measures, so greater convergence among measures was expected. It is suggested that future studies consider different reporting source for measuring outcome variables.

## Conclusions

The findings of our study showed that the PedsQL 4.0 has acceptable psychometric properties as shown by excellent internal consistency, high test–retest reliability, and negative moderate to strong convergent validity with a functional impairment scale (WFIRS-S). Therefore, it is a reliable and valid scale in measuring health-related quality of life in a healthy sample of Iranian adolescents. The PedsQL 4.0 would be a useful instrument for persons with lower levels of quality of life. Significant gender and age differences in quality of life should be considered as important factors in assessment of quality of life in adolescents. In addition, the four-factor structure of the PedsQL 4.0 was not supported—alternative factor structures (e.g., the PedsQL 4.0 second-order factor model consisting of physical functioning and psychosocial health factors [24] and the five-factor model including physical, emotional, social, school, and missed school factors of the PedsQL 4.0 [35]) need to be studied in future research.

## Data Availability

The datasets used and/or analysed during the current study are available from the corresponding author on reasonable request.

## Abbreviations

PedsQL 4.0: Pediatric quality of life inventory version 4.0
WFIRS-S: Weiss Functional Impairment Rating Scale-Self-report
CFA: Confirmatory factor analysis
CFI: Comparative fit index
RMSEA: Root mean square error of approximation
ICC: Intraclass correlation coefficient
IRT: Item response theory
GRM: Graded response model
